# Microbial Biotransformation of Chicory by *Bacteroides fragilis*: In Vitro Implications for Obesity-Related Psoriasis

**DOI:** 10.3390/ijms262110428

**Published:** 2025-10-27

**Authors:** Arthur Chervet, Rawan Nehme, Clemence Defois-Fraysse, Caroline Decombat, Celine Auxenfans, Bertrand Evrard, Solene Michel, Edith Filaire, Jean-Yves Berthon, Assia Dreux-Zigha, Laetitia Delort, Florence Caldefie-Chezet

**Affiliations:** 1 Unité de Nutrition Humaine (UNH), CRNH-Auvergne, National Research Institute for Agriculture, Food and Environment (INRAE), Université Clermont-Auvergne, 63000 Clermont-Ferrand, Francecaroline.decombat@uca.fr (C.D.); bertrand.evrard@uca.fr (B.E.); solene.michel@uca.fr (S.M.); edith.filaire@uca.fr (E.F.); laetitia.delort@uca.fr (L.D.); florence.caldefie-chezet@uca.fr (F.C.-C.); 2 Greencell, Biopôle Clermont-Limagne, 63360 Saint-Beauzire, France; clemencefraysse@greencell.tech (C.D.-F.); jeanyvesberthon@greentech.fr (J.-Y.B.); assiadreux@greencell.tech (A.D.-Z.); 3 Banque de Tissus et de Cellules, Hôpital Edouard-Herriot, 69000 Lyon, France; celine.auxenfans@chu-lyon.fr

**Keywords:** *Bacteroides fragilis*, fermentation, prebiotics, postbiotics, obesity, inflammation, psoriasis

## Abstract

Obesity, a global health crisis, is linked to chronic low-grade inflammation and an increased risk of developing various chronic diseases, including psoriasis. Probiotics, postbiotics, and fermented foods have shown promise in combating inflammation and obesity. This study aimed to develop and characterize a chicory extract fermented with *Bacteroides fragilis* (C-*B. fragilis*) and its supernatant (phyto-postbiotic supernatant, PPS) as potential treatments for obesity, inflammation, and psoriasis. Polyphenols, organic acids, and amino acids were identified in the metabolic profile of C-*B. fragilis*. PPS and C-*B. fragilis* extract both revealed potent anti-inflammatory, anti-obesity, and antioxidant activities. In vitro assays highlighted that PPS significantly reduced the production of reactive oxygen species (ROS), the expression of pro-inflammatory cytokines (TNF-α, IL-6, IL-8) in macrophages, and the secretion of IL-1β in LPS-stimulated PBMCs. Moreover, PPS decreased triglyceride content in human adipocytes and modulated the expression of leptin and adiponectin. Regarding psoriasis, PPS reduced pro-inflammatory cytokines (IL-6, IL-1β) in both psoriatic keratinocytes and a co-culture model mimicking the skin-adipose tissue interface. In addition, PPS lowered S100 calcium-binding protein A7 (S100A7) expression in the co-culture model, suggesting a potential role in restoring skin barrier function. In summary, our results highlight the potential of PPS extract (supernatant of chicory fermentation by *Bacteroides fragilis*) as a promising therapeutic strategy for the management of obesity-related inflammation and psoriasis.

## 1. Introduction

Obesity, which has emerged as a global public health concern, is defined as an excessive accumulation of body fat that may impair health [1]. Obesity epidemiology shows that its incidence has gradually increased in recent decades [2]. According to WHO data, more than 2.5 billion people were overweight by 2022, with more than 890 million being obese [3]. Obese adipose tissue is characterized by adipocyte hypertrophy and hyperplasia [4]. Moreover, the pathological adipose tissue microenvironment promotes the phenotypic switch of anti-inflammatory M2 macrophages toward a pro-inflammatory M1 profile, accompanied by the recruitment of additional pro-inflammatory immune cell populations. Pro-inflammatory immune cells produce cytokines such as tumor necrosis factor alpha (TNF-α), interleukin (IL)-6, and interferon gamma (IFN-γ) [5]. Meanwhile, hypertrophic adipocytes, which are immunocompetent cells, can release chemokines, pro-inflammatory cytokines, and adipokines, notably leptin, a pro-inflammatory adipokine [6]. This local inflammation becomes self-sustaining, resulting in chronic, low-grade inflammation. This condition contributes to the development of several pathologies, including cardiovascular disease, cancer, and immune-driven skin disorders like psoriasis [7].

Psoriasis is a chronic inflammatory disease that affects roughly 2 to 3% of the world’s population. The pathology is marked by excessive epidermal hyperplasia, pronounced infiltration of immune effector cells into the cutaneous tissue, and a sustained local inflammatory response [8]. Immunopathogenesis of psoriasis is mediated by dendritic cells (DCs), T cells, and keratinocytes. Activated DCs release TNF-α, IL-12, and IL-23, which help sustain or promote Th1, Th17, and Th22 cells. These cells release IL-17, IFN-γ, and IL-22, which promote keratinocyte proliferation and the production of inflammatory mediators like chemokines, cytokines, and antimicrobial peptides (cathelicidin, β-defensins, and S100 proteins) [9]. In addition to this immunological cascade, several microorganisms (including *Staphylococcus aureus*, *Streptococcus pyogenes*, and *Candida* spp.) have been identified as triggering or exacerbating factors in psoriasis. Their presence may modulate the local immune response and contribute to the chronicity of the disease [10,11].

A relationship between obesity and psoriasis has also been established [12]. Obese people are more likely to acquire psoriasis, and their plasma leptin levels are higher. This high concentration has a positive correlation with body mass index (BMI), psoriasis area and severity index (PASI scores), and is inversely correlated with adiponectin levels. Leptin has been demonstrated to modulate DCs and helper T cells, key players in psoriasis immunopathogenesis [13]. Furthermore, it increases the production of pro-inflammatory cytokines, which leads to keratinocyte proliferation [14].

*Bacteroides fragilis* (*B. fragilis*) is a Gram-negative anaerobic bacterium and a key member of the human gut microbiome. It comprises enterotoxigenic (ETBF) and non-toxigenic (NTBF) strains. While its role in psoriasis and obesity remains unexplored, NTBF strains have shown anti-inflammatory effects in vitro and in vivo, notably through modulation of cytokines such as IL-10, IL-1β, and IL-6, and downregulation of the NF-κB pathway [15,16].

The aim of our study was to develop and evaluate an extract obtained by fermenting a prebiotic (chicory) with a non-toxigenic *B. fragilis* strain. We selected chicory because, in a subsequent study, extracts from horse chestnut fermentation were less effective than chicory extracts cultured with other bacteria at lowering ROS generation. Furthermore, the selection of *Bacteroides fragilis* was primarily driven by its well-recognized ability to modulate inflammation, notably through the secretion of Polysaccharide A (PSA) [17]. In addition, numerous recent studies have identified it as a “Next-Generation Probiotic” due to its promising functional properties [18,19]. Finally, this strain was selected following a preliminary screening of eleven bacterial strains derived from the human gut microbiota. Among the various combinations tested, the fermentation of chicory by *B. fragilis* demonstrated the most significant effects in reducing oxidative stress. In this follow-up study, we also examined horse chestnut extract fermented by *B. fragilis*, though the results did not yield conclusive evidence [20]. The idea was to test a first extract composed of inactivated bacteria and metabolites produced during fermentation, and a second extract composed only of the produced metabolites. We hypothesized that the anti-inflammatory effects would occur at the keratinocyte and adipocyte levels. By acting in a multifactorial manner on several components involved in the development and exacerbation of psoriasis—notably obesity through adipose tissue, low-grade systemic inflammation, and directly on keratinocytes—these fermented extracts studied could offer an innovative and integrated approach to improving the management of obesity-related psoriasis.

## 2. Results

### 2.1. Metabolite Identification in C-B. fragilis Extract

**Figure 1:**
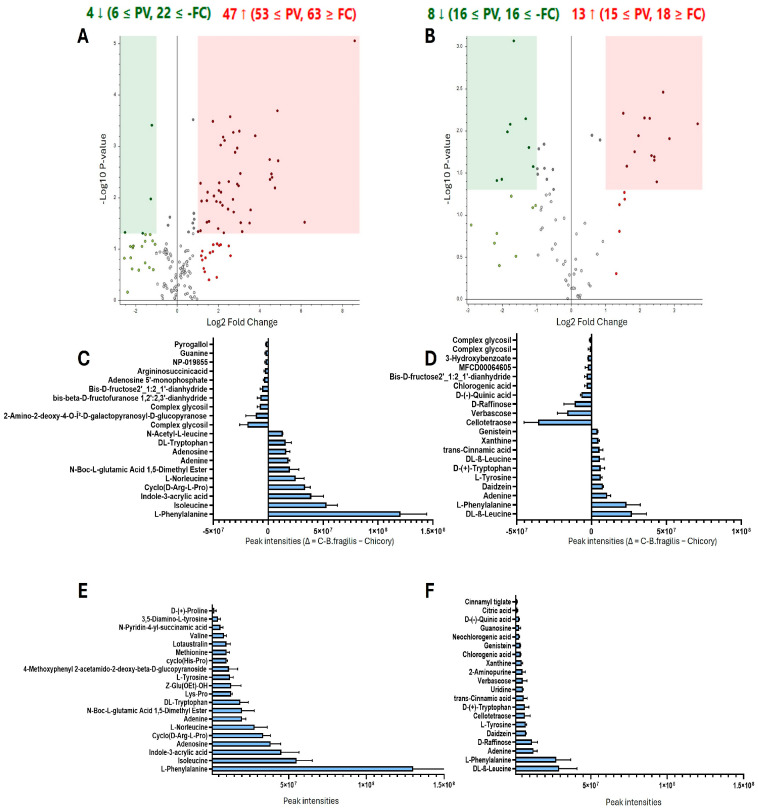
Untargeted metabolomics. Volcano plots showing the comparison of C-*B. fragilis* and Chicory extract in the positive (**A**) and negative (**B**) ion modes. The differential presence of the metabolites was determined as a fold-change ratio (C-*B. fragilis* extract/Chicory extract, >1 or <−1, *p* < 0.05 multivariate paired *t*-test). Each point represents a metabolite. Red dots represent upregulated metabolites, and green dots represent downregulated ones. The metabolite peak intensity between the C-*B. fragilis* extract and the chicory extract, expressed as delta (Δ = C-*B. fragilis* − Chicory) in positive (**C**) and negative (**D**) ion modes, represent which metabolites were primarily generated and which were consumed. Top 20 metabolites with the highest peak intensity in C-*B. fragilis* extract in the positive (**E**) and negative (**F**) ion modes.

In order to identify which metabolites were differentially present in group C-*B. fragilis* (and PPS that includes) and Chicory, a non-targeted metabolic analysis was performed using positive and negative ions. The volcano plots in Figure 1A,B showed the global distribution of metabolites. The differential expression was determined based on fold-change ratio (C-*B. fragilis* extract vs. chicory extract) (>1 or <−1, *p* < 0.05, multivariate test). In the positive ionization analysis (Figure 1A), 51 metabolites were shown to be differentially regulated, 4 to the lower and 47 to the higher. The negative ionization analysis (Figure 1B) revealed the presence of 21 different metabolites, 8 of which were regulated downward and 13 of which were regulated upward. However, this approach is based on ratios, and many metabolites may be completely consumed or simply created. In Figure 1C,D, we used the difference between the metabolites found after fermentation and before fermentation (Δ = C-*B. fragilis* extract–chicory extract) to determine which metabolites were primarily generated and which were consumed. We observe that the main metabolites produced are amino acids (L-phenylalanine, isoleucine, adenine, tryptophan) and acids (indole-3-acrylic acid, trans-cinnamic acid). We noticed the formation of daidzein and genistein. Sugars are the primary compounds used by bacteria. In fact, many sugars (verbascose, raffinose, cellotetraose, glucopyranose, pyrogallol, fructose) have a strong negative intensity, indicating that they were more abundant prior to fermentation. To characterize the C-*B. fragilis* extract, we compiled a list of the 20 most intense peaks (Figure 1E,F) and demonstrated that the C-*B. fragilis* extract contained a variety of amino acids (BCAAs, aromatic amino acids), polyphenols, and specific acids (indole-3-acrylic acid, daidzein). To investigate the production of short-chain fatty acids (SCFAs), HPLC analysis was performed. Propionic and butyric acids were not found, but acetic acid was, at a concentration of 3.08 g/L. Furthermore, lactic acid (5.38 g/L), citric acid (3.75 g/L), malic acid (2.14 g/L), and succinic acid (2.10 g/L) were produced as a result of the fermentation process (Table S1).

### 2.2. C-B. fragilis Extract and Its Supernatant (PPS) Inhibited ROS Production by Blood Leukocytes

**Figure 2:**
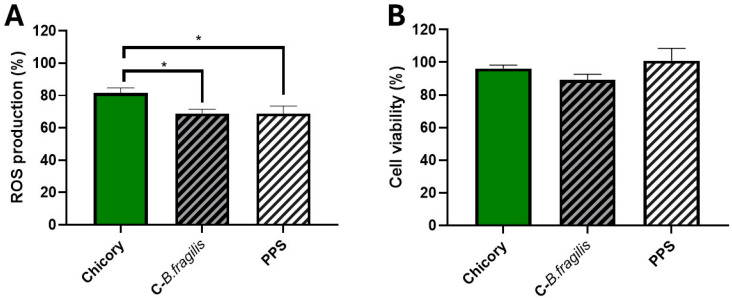
Effects of C-*B. fragilis* and PPS on ROS production by blood stimulated-leukocytes and their viability. Cells were incubated with C-*B. fragilis*, PPS or the control prebiotic (50 µg/mL) and stimulated with PMA (1 µM). After 2 h, we measured ROS production (**A**) and cell viability by a resazurin test (**B**). Data were expressed as mean ± SEM (Control = 100%) and analyzed using one-way ANOVA followed by Tukey’s post hoc test. Differences were considered significant at *p* < 0.05. * *p* < 0.05. (n = 10–12).

We investigated the antioxidant effect of C-*B. fragilis*, a combination of chicory fermented by *B. fragilis* and its supernatant (phyto-postbiotic supernatant; PPS), on PMA-induced ROS production in blood leukocytes (at 50 µg/mL). We aimed to determine whether our combinations exerted a stronger antioxidant effect compared to chicory alone. Incubation of leukocytes with C-*B. fragilis* or PPS significantly inhibited ROS production compared to chicory (Figure 2A) (−31 ± 7%, −31 ± 12%, respectively, vs. −18 ± 10% for chicory, *p* < 0.05). No difference was noted between C-*B. fragilis* and PPS. This effect was not due to a decrease in cell viability (Figure 2B).

### 2.3. PPS Decreased the Expression of Genes Involved in M1-like Macrophage Polarization

**Figure 3:**
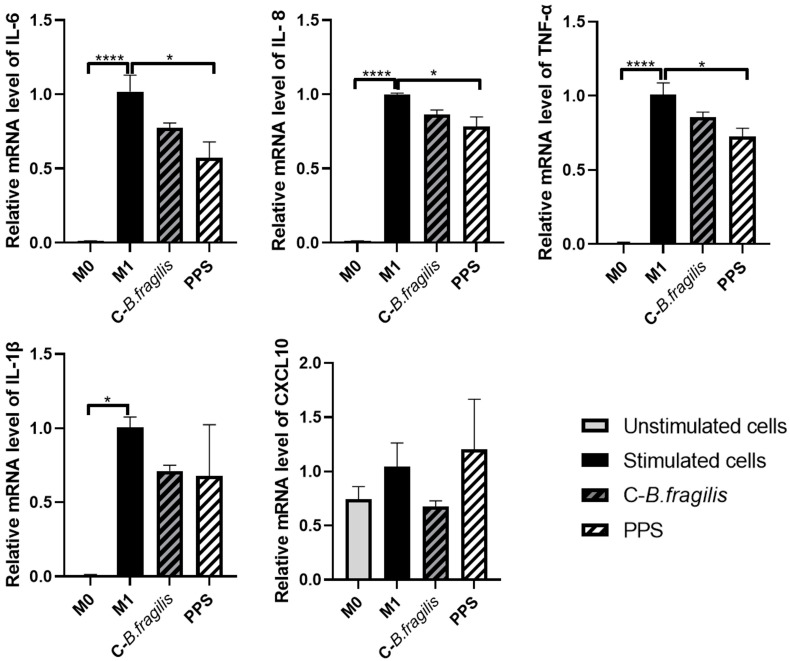
Effects of C-*B. fragilis* and PPS on macrophage polarization. THP-1 cells were first activated with 10 µM PMA, followed by stimulation with LPS and IFNγ to induce M1 polarization. The effects of C-*B. fragilis* and PPS extract (50 µg/mL) were assessed at each stage of the polarization process. Gene expression was quantified by real-time qPCR and normalized using *GAPDH* as an internal control. Data were expressed as mean ± SEM and analyzed using one-way ANOVA followed by Tukey’s post hoc test (n = 3). Differences were considered significant at *p* < 0.05. * *p* < 0.05, **** *p* < 0.0001.

To assess the potential effects on macrophages, another key player in innate immunity, THP1 cells were treated with C-*B. fragilis* or PPS (50 µg/mL) during the polarization of M0 macrophages into pro-inflammatory M1 macrophages (Figure 3). Only the PPS treatment resulted in a significant decrease in the expression of *IL-6* (RQ = 0.57 ± 0.17, *p* < 0.05), *IL-8* (RQ = 0.78 ± 0.09, *p* < 0.05), and *TNF-α* (RQ = 0.72 ± 0.07, *p* < 0.05). Although treatment with C-*B. fragilis* seemed to induce decreases in the expression of *IL-6*, *IL-8*, *TNF-α*, *IL-1β*, and *CXCL10*, none of these decreases were statistically significant.

### 2.4. C-B. fragilis and PPS Extract Modestly Decreased IFNγ, IL-17A and IL-1β Secretion in LPS-Stimulated PBMCs

**Figure 4:**
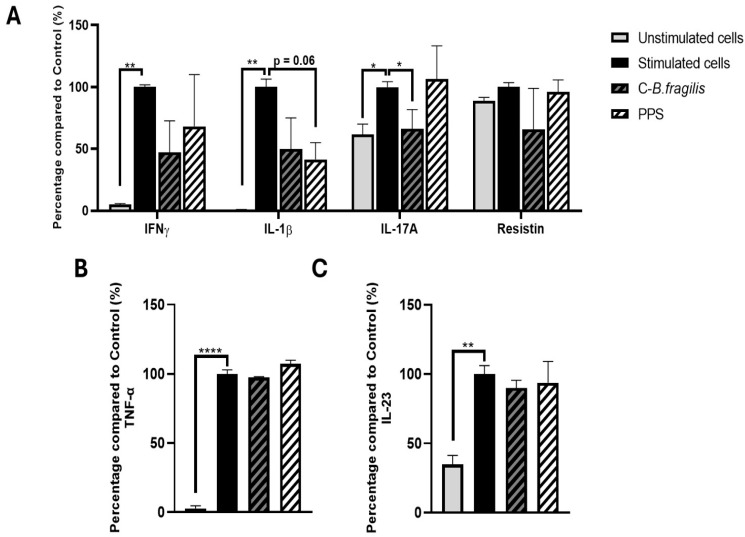
Effects of C-*B. fragilis* and PPS on LPS-stimulated PBMCs. Cells were incubated for 24 h with LPS (10 µg/mL) and either C-*B. fragilis* or PPS (0 or 50 µg/mL). (**A**) Cytokine concentrations were quantified using the Luminex Bio-Plex 200 System, following the manufacturer’s protocol with optimized standards and antibody concentrations. (**B**) TNF-α levels measured by ELISA assay, (**C**) IL-23 levels measured by ELISA assay. Data are expressed as mean ± SEM (Stimulated cells = 100%) and analyzed using one-way ANOVA followed by Tukey’s post hoc test (n = 4). Differences were considered significant at *p* < 0.05. * *p* < 0.05, ** *p* < 0.01, **** *p* < 0.0001.

To further investigate whether cytokine production was influenced by our treatments, PBMCs, stimulated or not with LPS, were treated with 50 µg/mL of C-*B. fragilis* or PPS. Treatment with C-*B. fragilis* induced a significant decrease in IL-17A (−34 ± 20%, *p* < 0.05) and non-significant decreases in IFNγ (−53 ± 31%) and IL-1β (−50 ± 32%) (Figure 4). Treatment with PPS only induced non-significant decreases in IL-1β (−59 ± 15%, *p* = 0.06). Treatment with either C-*B. fragilis* or PPS showed no significant effect on IL-23 and TNF-α production.

### 2.5. C-B. fragilis Affected the Expression of Leptin and Hormone-Sensitive Lipase (HSL) by Human Adipospheroids, and PPS Decreased Adipogenesis

**Figure 5:**
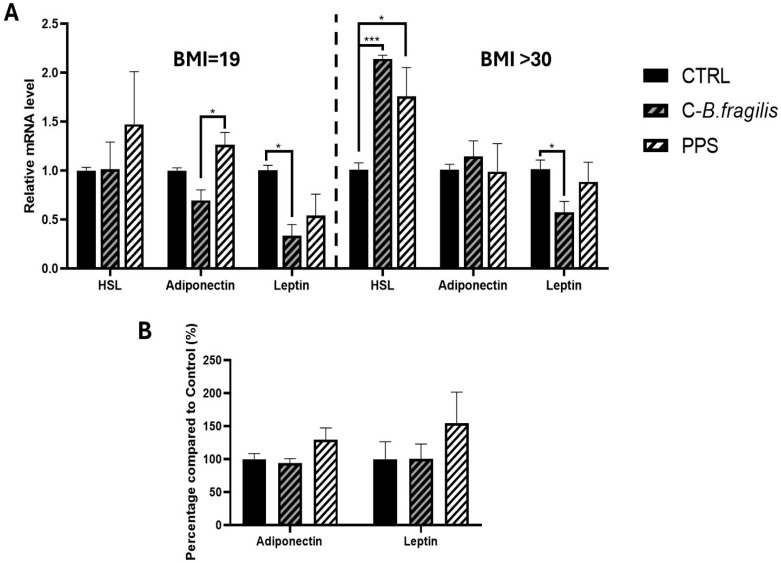
Effect of C-*B. fragilis* and PPS on adipokine modulation in human adipospheroids. Human adipospheroids were treated with 50 μg/mL of C-*B.fragilis* or PPS for 24 h. Total RNA isolated from untreated cells was used as the control. Data showed the relative mRNA expression of *HSL*, *Leptin*, and *Adiponectin* normalized to *GAPDH*. (**A**) *Leptin*, *adiponectin*, and *HSL* expression on adipospheroids from normal-weight women (BMI = 19) and from obese women (BMI > 30), (**B**) Leptin and adiponectin secretions by adipospheroids (BMI > 30) were measured by ELISA assay (Control = 100%). Date were expressed as mean ± SEM and analyzed using one-way ANOVA followed by Tukey’s post hoc test (n = 5). Differences were considered significant at *p* < 0.05. * *p* < 0.05, *** *p* < 0.001.

The effects of the extracts were subsequently evaluated on human adipospheroids composed of adipose cells from normal-weight (BMI < 20) and obese women (BMI > 30). C-*B. fragilis* significantly decreased *Leptin* expression in adipospheroids from normal-weight individuals (RQ = 0.33 ± 0.14, *p* < 0.05) and obese people (RQ = 0.57 ± 0.20, *p* < 0.05) (Figure 5A), whereas PPS had no significant effect. In adipospheroids from obese individuals, both treatments significantly increased *HSL* expression (RQ = 2.14 ± 0.06, *p* < 0.001 for C-*B. fragilis* and RQ = 1.76 ± 0.51, *p* < 0.05 for PPS). Both treatments had no effect on the secretion of *leptin* or *adiponectin* (Figure 5B).

**Figure 6:**
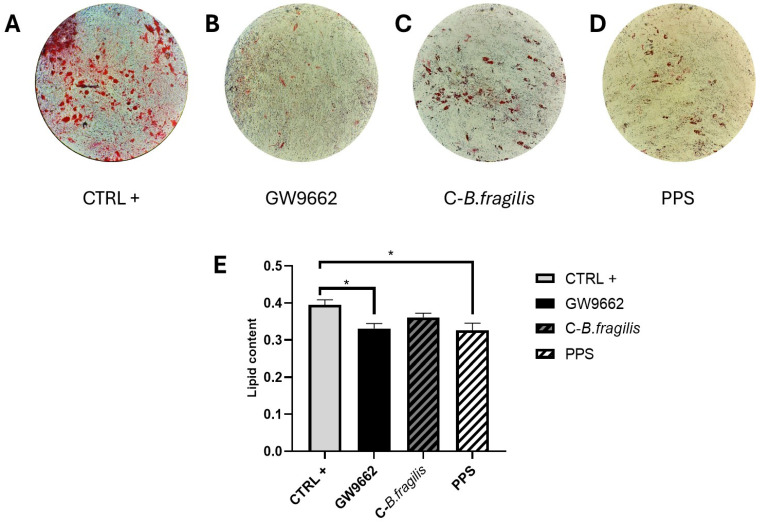
Effect of C-*B. fragilis* and PPS on reduction in intracellular triglyceride content in differentiated human adipocytes. (**A**) Differentiated human mature adipocytes (control condition); (**B**) differentiated mature adipocytes cultured with GW9662; (**C**) differentiated mature adipocytes in the presence of 50 μg/mL C-*B. fragilis*; (**D**) differentiated mature adipocytes in the presence of 50 μg/mL PPS; (**E**) quantification of Oil Red O staining intensity measured at 490 nm after 14 days of chronic exposure to 50 μg/mL of C-*B. fragilis*, PPS extract, or GW9662. Date were expressed as mean ± SEM and analyzed using one-way ANOVA followed by Tukey’s post hoc test (n = 3). Differences were considered significant at *p* < 0.05. * *p* < 0.05.

We next studied the potential effects of the extracts on obesity by examining their impact on adipogenesis. Microscopic study of Oil Red O-stained differentiated human mature adipocytes cultivated in the absence or presence of GW9662, a PPARγ agonist (Figure 6B), C-*B. fragilis* (Figure 6C), or PPS (Figure 6D) indicated intracellular lipid droplet formation. Chronic PPS treatment appeared to reduce lipid accumulation. Extracted Oil Red O staining revealed a 16% (*p* < 0.05) reduction in lipid content with GW9662 (negative control) and 17% (*p* < 0.05) with PPS compared to the control (Figure 6E), indicating a decrease in intracellular lipid accumulation.

### 2.6. C-B. fragilis and PPS Extract Affected the Expression and Secretion of Pro-Inflammatory Cytokines in Human Psoriatic Keratinocytes

**Figure 7:**
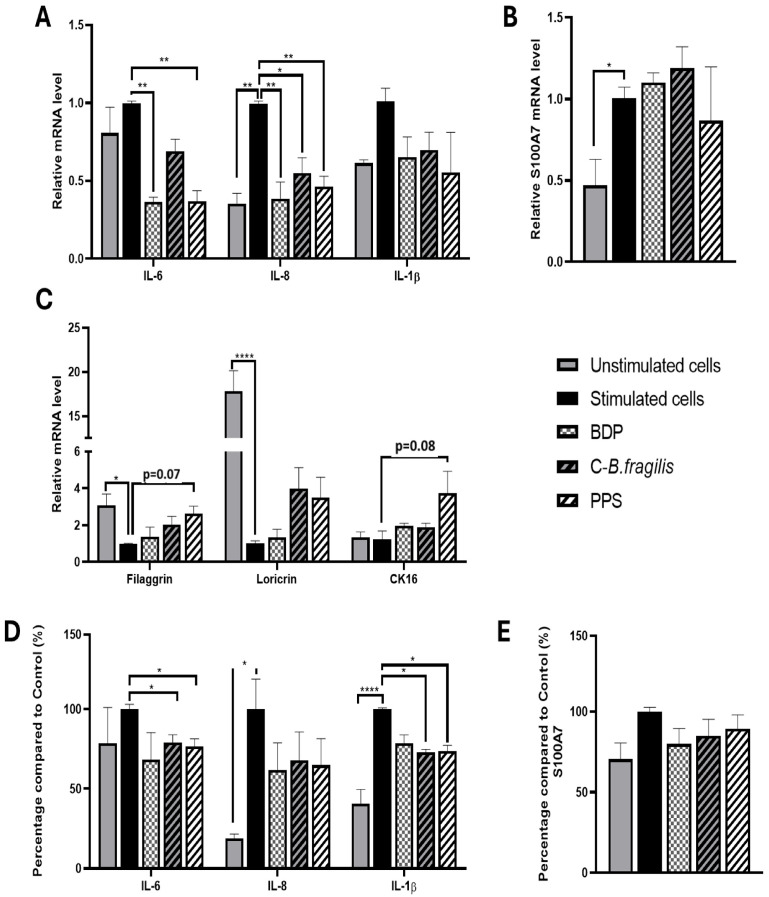
Effect of C-*B. fragilis* and PPS on human psoriatic keratinocytes. The monolayer was pre-treated with IL-17A (20 ng/mL), IL-22 (20 ng/mL), and TNF-α (5 ng/mL) for 24 h. After that, cells were washed with PBS, and fresh medium without stimulation was added for the treatment. After 24 h of treatment, the cells’ RNA and supernatant were collected. Gene expression was quantified by real-time qPCR and normalized using *GAPDH* as an internal control. (**A**) *IL-6*, *IL-8*, and *IL-1β* gene expression, (**B**) *S100A7* gene expression, (**C**) *Filaggrin*, *Loricrin*, and *CK16* gene expression, (**D**) Cytokine levels were measured with the Luminex Bio-Plex 200 System using optimal concentrations of standards and antibodies according to the manufacturer’s instructions, (**E**) S100A7 levels measured by ELISA assay. Data are expressed as mean ± SEM (Stimulated cells = 100%) and analyzed using one-way ANOVA followed by Tukey’s post hoc test (n = 3). Differences were considered significant at *p* < 0.05. * *p* < 0.05, ** *p* < 0.01, **** *p* < 0.0001.

To explore the potential effects of the extract on psoriasis, human keratinocytes from psoriatic biopsies were used. The treatment with C-*B. fragilis* significantly reduced *IL-8* expression (RQ = 0.54 ± 0.12, *p* < 0.05) but not its secretion (Figure 7A,D). Concerning IL-6, C-*B. fragilis* significantly decreased its secretion (−21 ± 6%, *p* < 0.05), whereas PPS was able to reduce both its expression (RQ = 0.37 ± 0.09, *p* < 0.01) and secretion (−24 ± 6%, *p* < 0.05). C-*B. fragilis* and PPS significantly reduced the expression of IL-8 (RQ = 0.54 ± 0.12, *p* < 0.05 for C-*B. fragilis*, and RQ = 0.46 ± 0.08, *p* < 0.01 for PPS) but not its secretion. On the contrary, the two extracts significantly decreased the secretion of IL-1β (−27 ± 2%, *p* < 0.05 for C-*B. fragilis* and −26 ± 5%, *p* < 0.05 for PPS) but not its expression.

Concerning *S100A7*, a biomarker for psoriasis (playing a role in the inflammation and abnormal skin growth), no significant change in expression or secretion was observed regardless of treatment (Figure 7B,E).

Concerning epidermal barrier proteins, only PPS treatment enhanced gene expression but not significantly (*filaggrin*: RQ = 2.63 ± 0.48, *p* = 0.07; cytokeratin 16 (*CK16*): RQ = 3.74 ± 1.56, *p* = 0.08) (Figure 7C).

### 2.7. Effect of C-B. fragilis and PPS Extract on the Interaction Between Adipocyte/Psoriatic Keratinocytes in a Co-Culture Model

**Figure 8:**
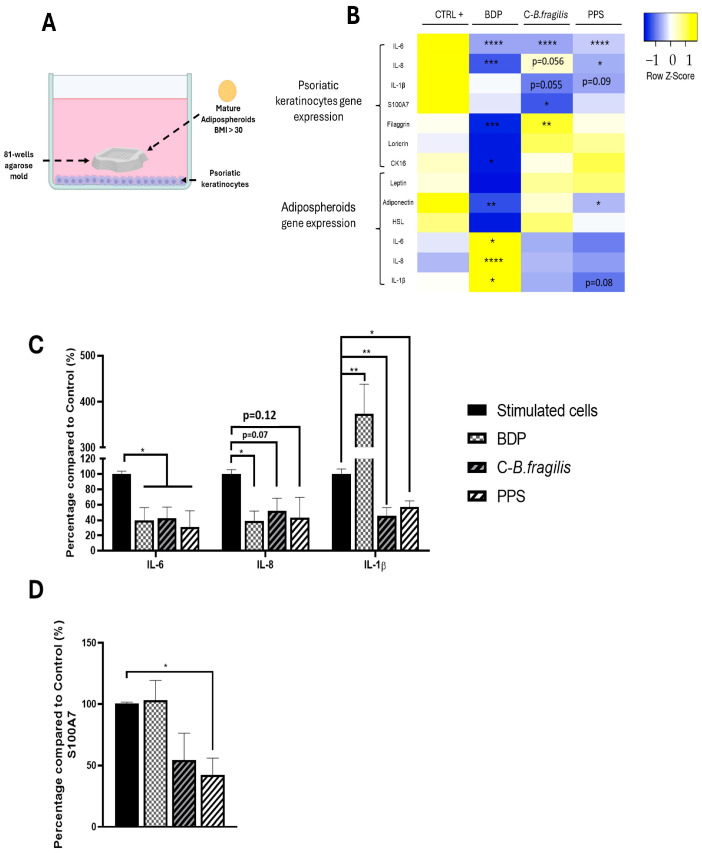
Effets of C-*B. fragilis* and PPS on Adipocyte/Psoriatic keratinocyte interactions. (**A**) Graphical representation of the co-culture system in which psoriatic keratinocytes (in the basal part of the well) were co-cultured with adipospheroids in agarose molds; (**B**) Heatmap representing mRNA expression of *IL-6*, *IL-8*, *IL-1β*, *S100A7*, *Filaggrin*, *Loricrin*, and *CK16* by psoriatic keratinocytes and *Leptin*, *Adiponectin*, *HSL*, *IL-6*, *IL-8*, and *IL-1β* by human obese adipospheroids after 24 h of co-culture measured by qRT-PCR. Total RNA isolated from untreated psoriatic keratinocytes or adipospheroids was used as the control. Normalized expression values are z-score normalized for each gene (**C**). The secretion of 3 cytokines was measured with the Luminex Bio-Plex 200 System using optimal concentrations of standards and antibodies according to the manufacturer’s instructions. (**D**) The secretion of S100A7 was measured by ELISA assay. Data were expressed as mean ± SEM (Control = 100%) and analyzed using one-way ANOVA followed by Tukey’s post hoc test (n = 3) (vs. CTRL). Differences were considered significant at *p* < 0.05. * *p* < 0.05, ** *p* < 0.01, *** *p* < 0.001, **** *p* < 0.0001.

To simulate the impact of obese adipocytes on psoriatic keratinocytes, we developed a co-culture system in which psoriatic keratinocyte cells were cultured with human adipospheroids made of adipose cells from an obese patient differentiated in agarose mold as described previously (Figure 8A).

A heatmap was used to show the differences in gene expression between the two cell types. In psoriatic keratinocytes, both treatments resulted in a significant reduction in *IL-6* expression (RQ = 0.31 ± 0.07 for C-*B. fragilis* and RQ = 0.39 ± 0.07 for PPS) and secretion (−57 ± 18% for C-*B. fragilis* and −69 ± 28% for PPS, *p* < 0.05) (Figure 8B,C). Furthermore, PPS induced a significant decrease in *IL-8* (RQ = 0.54 ± 0.15, *p* < 0.05) expression levels but not in its secretion (Figure 8B,C). Both treatments resulted in a significant decrease in IL-1β secretion (−54 ± 13% for C-*B. fragilis*, *p* < 0.01 and −42 ± 9% for PPS *p* < 0.05) (Figure 8B,C). In contrast, C-*B. fragilis* significantly reduced *S100A7* expression (RQ = 0.49 ± 0.13, *p* < 0.05) but not its secretion (Figure 8B). However, the quantity of S100A7 has significantly decreased for PPS (−58 ± 18%, *p* < 0.05) (Figure 8D). In terms of the proteins involved in the barrier function, only C-*B. fragilis* increased significantly the expression of *filaggrin* (RQ = 1.57 ± 0.06, *p* < 0.01).

At the adipocyte level, the treatment with PPS caused a significant decrease in the expression of *Adiponectin* (RQ = 0.60 ± 0.02, *p* < 0.05), whereas C-*B. fragilis* had no effect on its expression. PPS only reduced but not significantly *IL-1β* expression (RQ = 0.46 ± 0.11, *p* = 0.08) (Figure 8B).

## 3. Discussion

Obesity appears to be a risk factor for psoriasis, aggravates existing psoriasis, and can increase psoriasis severity in overweight people. Several studies have revealed a correlation between obesity and psoriasis [21,22,23]. The precise mechanisms linking obesity and psoriasis are not fully understood. Furthermore, excess weight can hinder the effectiveness of psoriasis medications [21]. Obesity is associated with increased susceptibility to cutaneous infections of both bacterial and fungal origin, which are recognized as contributing factors in the initiation and exacerbation of psoriatic manifestations [24,25]. Fermented foods, probiotics, and postbiotics offer promising perspectives in human health in the fight against inflammation and obesity [26,27,28]. Although less common, a few studies have investigated the therapeutic effects of probiotics—primarily Lactobacillus and Bifidobacterium strains—and postbiotics in the treatment of psoriasis [29,30]. The gut bacterium *B. fragilis* has been associated with reduced inflammation and improved metabolic health [16,31]. Additionally, *B. fragilis* is recognized for secreting a complex sugar molecule known as polysaccharide A (PSA). This is a key component of the bacterial capsule and has been shown to possess significant immunomodulatory properties [17].

Initially, our research focused on the development and characterization of fermented extracts. We then investigated the effects of these extracts on major cellular components implicated in obesity and chronic inflammation: adipocytes and macrophages. In parallel, we assessed their impact on keratinocytes, epithelial cells central to psoriasis pathogenesis. We hypothesized that *B. fragilis*-fermented chicory would produce health-promoting metabolites. Fermentation resulted in acetate production but lacked butyrate and propionate. Additionally, we observed increased levels of organic acids (malic, lactic, citric, and succinic) and amino acids (BCAAs and aromatics) in the fermented chicory. The ability of *Bacteroides fragilis* to generate short-chain fatty acids, organic acids, and amino acids has been well established in prior studies [32]. However, SCFA and organic acid production vary depending on the *Bacteroides fragilis* strain and the culture conditions employed [33].

Obesity induces metabolic stress, which contributes to the production of reactive oxygen species (ROS) [34]. ROS production is pivotal in the advancement of inflammatory diseases, serving as both signaling molecules and inflammatory mediators [35]. Numerous in vitro and in vivo studies have demonstrated the antioxidant potential of specific probiotic strains, notably Lactobacillus and Bifidobacterium species [36,37]. However, just one study focused on the antioxidant properties of *B. fragilis* and its culture supernatant. Cang et al. observed an antioxidant effect of the culture supernatant as well as the intracellular cell-free extract from *B. fragilis* [31]. Our data suggest that treatment with C-*B. fragilis* and PPS could attenuate ROS generation in PMA-stimulated leukocytes. Additionally, inactivated *B. fragilis* did not appear to augment ROS reduction. Notably, the PPS extract exhibited antioxidant activity similar to that observed with the C-*B. fragilis* extract. The oxidative stress in adipose tissue favors the infiltration and activation of M1 macrophages [38]. The substantially higher ROS production in M1 polarized macrophages relative to M2 macrophages serves as a positive feedback loop [39]. By activating signaling pathways like nuclear factor kappa B (NF-κB) and mitogen-activated protein kinase (MAPK), ROS regulate the function and phenotype of M1 macrophages [39,40]. Studies examining the effects of *Bacteroides fragilis* and its culture supernatant on macrophage polarization remain scarce and have produced variable outcomes across strains. In fact, a study by Chen et al. demonstrated that extracellular vesicles derived from *B. fragilis* induced polarization of RAW264.7 cells towards an M2 phenotype [41]. Deng et al. further showed that exposure to *Bacteroides fragilis* (strain ZY-312) or its cell lysate upregulated the expression of M1 surface markers CD80 and CD86 in bone marrow-derived macrophages [42]. Our findings indicate that PPS extract downregulates the expression of TNF-α, IL-6, and IL-8 in M1-polarized macrophages, suggesting a reduction in polarization toward the M1 phenotype. Additionally, PPS treatment decreased IL-1β production in LPS-stimulated PBMCs, while C-*B. fragilis* treatment led to reduced IL-17A secretion. Despite previous in vivo and in vitro studies demonstrating effects of live, inactivated bacteria, culture supernatant, or PsA, our data did not show any influence on TNF-α release [15,16]. Our findings suggest that the antioxidant and anti-inflammatory effects of our extracts are likely mediated by fermentation metabolites, rather than the inactivated *B. fragilis* itself. The modulation of the NF-κB, nuclear factor erythroid 2-related factor 2 (NRF2), and AMP-activated protein kinase (AMPK) pathways by these metabolites may underlie these observed properties. This hypothesis is corroborated by the in vitro study conducted by Wang et al., which demonstrated that PsA treatment attenuated p65 phosphorylation and inhibited NF-κB signaling in LPS-stimulated primary hepatocytes [43]. Furthermore, Anan et al. demonstrated that aromatic amino acids and their derived metabolites attenuate IFNγ-mediated signaling in LPS-activated THP-1 monocytes and A549 pulmonary epithelial cells. These findings are consistent with a broader context in which amino acids are recognized for their intestinal anti-inflammatory properties, particularly through the suppression of NF-κB and the activation of the transcription factor Nrf2 and its antioxidant response elements (AREs) [44,45]. Emerging evidence indicates that lactic, acetic, succinic, malic, and citric acids may contribute to anti-inflammatory and antioxidant pathways [46,47,48,49,50]. Moreover, metabolites such as daidzein and genistein found in the extracts have been shown to exert antioxidant and anti-inflammatory effects through modulation of key signaling pathways.

These metabolites have been shown to suppress NF-κB and signal transducer and activator of transcription 1 (STAT-1) signaling while simultaneously activating the AMPK pathway [51].

Previous research has consistently shown that individuals affected by both obesity and psoriasis exhibit significantly increased circulating leptin levels, alongside a marked reduction in adiponectin, when compared to healthy individuals [52,53]. Subsequent studies have confirmed the positive impact of probiotics on obesity-related outcomes [54]. To date, no studies have been conducted to explore the direct role of *B. fragilis* in reducing obesity. Our findings demonstrate that treatment with C-*B. fragilis* extract resulted in a significant downregulation of adiponectin and leptin gene expression in human mature lean adipospheroids (BMI < 20), and leptin gene expression in human mature obese adipospheroids (BMI > 30). Although these alterations were detected at the mRNA level, corresponding protein expression remained unchanged. Moreover, PPS extract was shown to modulate adipogenesis. Specifically, PPS treatment led to a reduction in lipid accumulation within adipocytes during differentiation, suggesting a decreased differentiation of obese preadipocytes into mature adipocytes. These effects may be attributed to the potential modulation of transcription factors CCAAT/enhancer binding protein beta/delta (C/EBPβ/δ), C/EBPα, and peroxisome proliferator-activated receptors gamma (PPARγ) by fermentation-derived metabolites.

Keratinocytes play a pivotal role in the initiation and maintenance phases of psoriasis [9]. Being an integral component of the innate immune system, keratinocytes are capable of responding to a variety of stimuli [9]. Once activated, keratinocytes become highly proliferative and can produce numerous chemokines (CXCL1/2/3, CXCL8, CXCL9/10/11, CCL2, and CCL20), antimicrobial peptides (S100A7) to induce innate immunity, and pro-inflammatory cytokines (TNF-α, IL-6, IL-8, and IL-1β) [9]. Previous studies have demonstrated that both probiotics and postbiotics can attenuate cutaneous inflammation. Notably, Kim et al. reported that culture supernatant derived from fermented *Smilax china* L. leaves and Lactobacillus acidophilus effectively inhibited NF-κB activation in HaCaT cells stimulated with TNF-α and IFNγ [55]. In another study, Chung et al. demonstrated that treatment with culture supernatant from *Lactobacillus helveticus* and *Lactococcus lactis* (1:1) decreased IL-8 secretion in HaCaT cells co-cultured with live *Staphylococcus aureus* or *Cutibacterium acnes* [56]. In our study, we used betamethasone dipropionate (1 µM) as an anti-inflammatory control. Our results reveal that treatments with C-*B. fragilis* and PPS induce a decrease in IL-6, IL-8, and IL-1β expression, as well as IL-6 and IL-1β secretion, similar to BDP in psoriatic keratinocytes from patients. Furthermore, treatment with PPS induces an increase in filaggrin, a protein downregulated in psoriasis that plays a crucial role in the formation of the skin barrier. In a co-culture model combining mature obese adipospheroids and psoriatic keratinocytes, designed to evaluate the impact of adipokine secretion on keratinocyte behavior, we observed consistent effects on pro-inflammatory cytokines. Treatments with C-*Bacteroides fragilis* and PPS extract led to a reduction in the expression of IL-6, IL-8, and IL-1β. At the protein level, both treatments decreased IL-6 and IL-8 concentrations to a similar extent as BDP. Moreover, they reduced the amount of IL-1β compared to BDP, which increased its level in this model. Only C-*B. fragilis* extract induced an increase in filaggrin expression in this co-culture model. Furthermore, PPS treatment led to a decrease in *S100 calcium-binding protein A7* (S100A7) levels. Once again, the results were similar between the two treatments, suggesting that the metabolites produced during fermentation possess the biological activities. These results could be explained by the multi-cellular anti-inflammatory effects demonstrated in this study. Additionally, PPS may target signaling pathways involved in psoriasis, such as NF-κB, janus kinase/signal transducer and activator of transcription (JAK-STAT) and non-receptor tyrosine-protein Kinase (TYK2) pathways, MAPK pathway, and phosphoinositide 3-kinase/Akt (PI3K-AKT) pathway [9,57].

To the best of our knowledge, this is the first study to generate and characterize an extract derived from the fermentation of chicory by *B. fragilis*. In addition to using human immune cells, we also used mature human adipocytes organized into adispheroïds, which provided a unique and complementary in vitro tool to further investigate the mechanism of action. Indeed, most in vitro tests on adipogenesis and anti-obesity effects are performed on 3T3-L1, a murine cell line. Additionally, we used psoriatic keratinocytes derived from patient biopsies, which have a different transcriptome profile than human keratinocytes commonly used in in vitro experiments [58]. One significant finding of our research is the anti-inflammatory activity of the culture supernatant derived from chicory fermented by *B. fragilis*, as demonstrated by its effects on macrophage polarization and psoriatic keratinocytes. Furthermore, PPS extract has demonstrated powerful anti-adipogenic properties. One of the primary limitations of in vitro research lies in the challenge of translating findings to in vivo systems. To address this, future studies should investigate the topical application of our extract in murine models of imiquimod-induced psoriasis, as well as in obese mice with imiquimod-induced psoriasis, to assess both local and systemic effects. Additionally, mechanistic studies are required to elucidate the underlying mechanisms of action. Moreover, it would be relevant to investigate the impact of this extract on the skin microbiota, whose dysbiosis is well-documented in psoriasis. The ability to modulate the cutaneous microbiome could represent a complementary approach in the therapy of this condition. Through our research two chicory extracts fermented by *B. fragilis* are generated and characterized. Moreover, by addressing both inflammation and adipogenesis, our findings suggest that PPS extract may have potential as a therapeutic agent for obesity-related psoriasis pending in vivo and clinical validation. This model allowed us to investigate the effects of treatments on the inflammatory state of psoriasis and to study the communication between keratinocytes and adipocytes.

## 4. Materials and Methods

### 4.1. Generation of a Chicory Extract Fermented with Bacteroides fragilis (C-B. fragilis)

A chicory extract fermented with *Bacteroides fragilis* was developed. Briefly, *Bacteroides fragilis* (strain: DSM1396) was pre-cultured under anaerobic conditions. After 40 h of fermentation on the prebiotic medium containing chicory, the cultures were centrifuged. The filtered supernatants were mixed with maltodextrin, while the bacterial pellets were heat-inactivated (1 h 100 °C). The two fractions were then combined and freeze-dried [20]. A second extract was produced, recovering only the supernatant from the fermentation process, called phyto-postbiotic supernatant (PPS).

For the chicory extract, the roots of common chicory (Cichorium intybus) (France) were cut into 0.5 to 2 cm pieces after being dried. Then, 200 g of chicory were extracted in 2000 mL of boiling water and left for 12 h at room temperature. Filtration of the extract was carried out on a 15 µm filter. The extract was concentrated with a rotary evaporator (at 40 °C) to 9.24% of dry matter (dm), containing 61.6%/dm of sugars (analyzed under the monosaccharide form after hydrolysis) [20].

### 4.2. Untargeted Metabolomic

Briefly, a 50 mg aliquot of C-*B.fragilis* extract was prepared and analyzed by high-resolution LC-MS (Orbitrap Fusion Lumos, Thermo Fisher Scientific, Waltham, MA, USA) in positive and negative modes. Separation was performed on a C18 column with an elution gradient using water/formic acid or ammonium acetate/acetonitrile mobile phases. Data acquisition was performed in AcquireX mode, combining precursor mass detection and iterative fragmentation. Detected compounds were identified using the mzCloud, ChemSpider, and Natural Product Atlas databases, with similarity scores and formula prediction calculated. Metabolomic analysis was performed on three separate C-*B.fragilis* extracts (n = 3), and the data presented are the mean of these biological replicates [20].

### 4.3. Blood Leukocyte Preparation

Blood was collected from healthy human volunteers (12 volunteers, Etablissement Français du Sang, EFS, Clermont-Ferrand, France). In compliance with sections L1222-1, L1222-8, L1243-4, and R1243-61 of the French Public Health Code, donors provided written informed consent for the use of their blood samples for research purposes under EFS contract no. 16-21-62. Hemolytic shock was used to obtain whole blood leukocytes using ammonium chloride solution (NH4Cl 115 µM; NaHCO3 12 µM, EDTA 0.01 µM). The cells were then centrifuged for 10 min at 1300 rpm and suspended in supplemented Roswell Park Memorial Institute 1640 Medium (RPMI-1640, Gibco, Thermo Fisher Scientific, Waltham, MA, USA) [59]. Supplements included fetal bovine serum (FBS, 10%) (Eurobio Scientific, Saclay, France), gentamicin (50 μg/mL), and glutamine (Gln, 2 mM), all from Thermo Fisher Scientific.

### 4.4. Peripheral Blood Mononuclear Cells (PBMCs) Preparation from Human Blood

Peripheral blood mononuclear cells (PBMCs) were isolated from buffy coats of three healthy donors (EFS, Clermont-Ferrand, France) using Ficoll-Histopaque^®^ 1077 (Sigma-Aldrich, St. Louis, MO, USA) density gradient centrifugation. After removal of plasma and collection of the mononuclear cell layer, erythrocytes were eliminated by hemolysis with ammonium chloride. Cells were washed and resuspended in complete RPMI medium (10% FBS, 50 µg/mL gentamicin, 2 mM glutamine), and adjusted to a final concentration of 1 × 10^6^ cells/mL for downstream experiments.

### 4.5. Reactive Oxygen Species (ROS) Generation by Leukocytes

Leukocyte preparations (n = 10–12) were made in accordance with the above instructions. In 96-well plates, 10^6^ cells/mL were cultured in complete medium with C-*B. fragilis* (50 µg/mL) or PPS (50 µg/mL) and dihydrorhodamine 123 (Dhr 123, 1 µM, Sigma-Aldrich), with or without 1 µM PMA stimulation for a duration of 120 min. The fluorescence intensity of rhodamine 123s, which results from the oxidation of Dhr 123, was measured using the Tecan Spark^®^ (Männedorf, Switzerland) every 5 min for 120 min (excitation/emission: 485/535 nm). The percentage of ROS produced by treated cells after stimulation relative to untreated cells (100%) was used to express the results.

### 4.6. Leucocyte Viability

10^6^ cells/mL were suspended in complete medium and treated with C-*B. fragilis* (50 µg/mL) or PPS (50 µg/mL), PMA (0 or 1 µM), and resazurin (25 µg/mL) in 96-well plates [59]. The Tecan Spark^®^ was used to measure fluorescence (excitation/emission: 544/590 nm) after two hours. The results were presented as the percentage of stimulated treated cells that remained viable as compared to stimulated untreated cells.

### 4.7. Determination of Cytokine Concentrations

PBMCs (10^6^ cells/mL) from three volunteers were incubated with or without lipopolysaccharide (LPS) and C-*B. fragilis* or PPS at a concentration of 50 µg/mL for 24 h. The supernatants were subsequently collected to assess cytokine secretion (IFNγ, IL-17A, IL-1β and Resistin) using Human Custom ProcartaPlex assays (Invitrogen™ ThermoFisher Scientific, Waltham, MA, USA). Cytokine levels were quantified utilizing optimal concentrations of standards and antibodies in accordance with the manufacturer’s guidelines. Following the completion of all assay steps, plates were analyzed using the Luminex Bio-Plex 200 system (Biorad, Marnes-la-Coquette, France), and data were processed with BioPlex Manager™ 4.1 software employing five-parameter logistic regression (5PL) curve fitting. The results were expressed as a percentage of stimulated treated cells vs. stimulated untreated cells (100%).

### 4.8. Human Monocytic Leukemia Cells

The human monocytic leukemia cell line THP-1 (American Type Culture Collection ATCC, TIB-202TM) was cultivated in RPMI supplemented with 10% FBS, 2 mM Gln, and 50 µg/mL gentamicin at 37 °C in a humidified environment of 5% CO_2_. For macrophage activation, THP-1 cells (4 × 10^5^ cells/mL) were cultured in 6-well plates for three days in a complete growth medium that contained 16.2 nM phorbol 12-myristate 13-acetate (PMA, Sigma-Aldrich). Following a 24 h incubation period with 10 pg/mL of LPS (Sigma-Aldrich) and 20 ng/mL of IFNγ (Gibco), they were polarized into M1-like macrophages [59] (n = 3).

### 4.9. Adipose Cell Culture

Preadipocyte cells were obtained from anonymous, healthy donors who were undergoing cosmetic surgery and had no underlying medical issues, in compliance with the Helsinki Declaration. Surgical residue was collected in compliance with French rules, which required patients to provide written informed consent and submit a statement to the Research Ministry (DC no. 2008162). Cells were isolated from women with obesity (BMI > 30) or normal weight (BMI = 19). To differentiate preadipocytes (PAs) into mature adipocytes (MAs), cells were seeded at confluence (33,500 cells/cm^2^) in a differentiation medium consisting of Dulbecco’s modified Eagle medium (DMEM/F12, Gibco, ThermoFisher Scientific, Carlsbad, CA, USA) supplemented with FBS (10%), Gln (1%), dexamethasone (980 mg/mL), T3 (6.5 mg/mL), hydrocortisone (25 mg/mL), insulin (3.5 mg/mL), rosiglitazone (1.78 mg/mL), isobutyl-methylxanthine (IBMX) (100 mg/mL, only for the first 3 days), and gentamycin (50 mg/mL) (Sigma-Aldrich, St. Louis, MO, USA). The medium was replaced every two days. Mature adipocytes were obtained after 14 days of differentiation.

### 4.10. Quantification of Lipid Accumulation

Pre-adipocytes were cultured for 14 days in differentiation medium (as described above). Every two days, the differentiation medium was changed. Treatments were added to this differentiation medium, either C-*B. fragilis* (50 µg/mL), PPS (50 µg/mL) or GW9662. At the end of the treatment, cells were washed twice with PBS before fixation with 4% paraformaldehyde (30 min at room temperature). After removal of the paraformaldehyde solution, cells were washed twice with water before being incubated with a 60% isopropanol solution for 5 min, then with the working Oil Red O solution (0.2% in 60% isopropanol) for 20 min. After 5 more washes with water, the fixed dye was redissolved in a fixed volume of 100% isopropanol in order to quantify the staining, and the optical density was measured at 490 nm using the microplate fluorometric reader (Tecan Spark^®^).

### 4.11. Generation of Adipospheroids

To make agarose molds, 20 g/L agarose (ThermoFisher Scientific) was combined with 0.9% *w*/*v* NaCl (Sigma-Aldrich), sterilized for 20 min at 120 °C, and placed in MicroTissues^®^ 3D Petri Dishes^®^ (81 wells, Sigma-Aldrich) according to the manufacturer’s instructions [60]. Cell attachment is inhibited in these molds, thus cells spontaneously aggregate to form spheroids via increasing intercellular adhesion molecules. Next, 200,000 preadipocytes/agarose mold were plated and cultured in DMEM/F12 medium (supplemented with 10% FBS and 1% Gln) at 37 °C in 5% CO_2_, yielding 81 potential adipospheroids per agarose mold. On the second day, the differentiation medium was applied as previously reported to produce mature adipocyte spheroids referred as adipospheroids.

Human preadipocytes were differentiated into spheroids over 8 days and then treated with C-*B. fragilis* or PPS (50 µg/mL) for 24 h. Gene expression of adipokines and *HSL* was assessed.

### 4.12. Human Psoriatic Keratinocyte Culture

Human psoriatic keratinocytes were purchased from CTI Biotech (Meyzieu, Lyon, France). Cells were seeded at a concentration of 7.5 × 10^4^ cells/well in a 12-well plate. Cells were cultured in serum-free DermaCult™ Keratinocyte Expansion Medium (StemCell Technologies, Vancouver, BC, Canada) at 37 °C in an incubator with 5% CO_2_. The monolayer was pre-treated with IL-17A (20 ng/mL), IL-22 (20 ng/mL), and TNF-α (5 ng/mL) during 24 h. After washing the cells with PBS, they were exposed to fresh medium without stimulation but with treatment (C-*B. fragilis* extract and phyto-postbiotic supernatant (PPS) at 50 µg/mL) or betamethasone dipropionate, which was used as an anti-inflammatory control (1 µM, Sigma-Aldrich). After 24 h, cell supernatants were collected for protein analysis with Luminex™ technology and S100A7 ELISA assay. Moreover, cells were collected to perform gene expression analysis with qRT-PCR.

### 4.13. Co-Culture Between Adipocytes and Psoriatic Keratinocytes

A co-culture system between psoriatic keratinocytes and adipospheroids was used as described above. Briefly, psoriatic keratinocytes were seeded at the bottom of the wells (7.5 × 10^4^ cells/well) and stimulated with IL-17A (20 ng/mL), IL-22 (20 ng/mL), and TNF-α (5 ng/mL) for 24 h. In parallel, adipospheroids were differentiated in agarose molds as described above, and co-cultured psoriatic keratinocytes, in a mixture of adipocyte differentiation medium and keratinocytes medium (50/50) in the presence of C-*B. fragilis* extract (50 μg/mL) or betamethasone dipropionate (1 µM). After 24 h of incubation, total RNA was extracted, RT-qPCRs were performed as previously described, and Luminex™ were performed on supernatant.

### 4.14. Real-Time Quantitative PCR (RT-qPCR)

Total RNA was extracted with TRIZOL reagent (Invitrogen, ThermoFisher Scientific). After the evaluation of the quantity and purity (Tecan Spark^®^), DNase treatment (DNase I Amplification grade, Invitrogen) and cDNA retro-transcription (HighCap cDNA RT Kit RNAse inhib, Invitrogen) were made according to the manufacturer’s recommendations. Amplification reaction assays were performed using SYBRGreen PCR Master Mix (Life Technologies, Thermo-Fisher Scientific) and primers designed by PrimerExpress software v3.0.1 (ThermoFisher Scientific) (Table 1) on a StepOneTM (Life Technologies). The expression of the following genes was measured: *GAPDH*, *IL1-β*, *TNF-α*, *IL-6*, *IL-8*, *CXCL10*, *LEPTIN*, *ADIPONECTIN*, *HSL*, *S100A7*, *LORICRIN*, *FILAGGRIN*, and *CK16*. Genes were considered significantly expressed and their transcript measurable if their corresponding Ct value was less than 35. Each sample was normalized to the endogenous reference gene (GADPH). The relative quantification method (RQ = 2^–ΔΔCt^) was used to calculate the relative gene expression of given samples with ΔΔCt = [ΔCt (sample1) − ΔCT (sample2)] and ΔCt = [Ct(target gene) − Ct(reference gene)].

### 4.15. ELISA Assays

ELISA assays for TNF-α, S100A7, IL-23 (ThermoFisher Scientific), adiponectin (R&D Systems, Minneapolis, MN, USA), and leptin (Abcam, Cambridge, UK) were performed following the manufacturers’ instructions.

### 4.16. Statistical Analysis

All the experiments were performed 3–12 times. Values are shown as mean ± SEM. Statistical significance between more than two groups was evaluated with a one-way ANOVA followed by Tukey’s post hoc test using GraphPad Prism software version 8 (GraphPad Software, San Diego, CA, USA). *p* < 0.05 was considered significant. Heatmap was plotted with Heatmapper (http://heatmapper.ca/ URL accessed on 10 June 2025)

## Figures and Tables

**Table 1:** PCR primers sequence.

Gene	Species	Forward Primer Sequence (5′-3′)	Reverse Primer Sequence (5′-3′)
*GAPDH*	Human	CACATGGCCTCCAAGGAGTAA	TGAGGGTCTCTCTCTTCCTCTTGT
*IL-8*	Human	CTGGCCGTGGCTCTCTTG	CCTTGGCAAAACTGCACCTT
*IL-1β*	Human	CCTGTCCTGCGTGTTGAAAGA	GGGAACTGGGCAGACTCAAA
*IL-6*	Human	GCTGCAGGCACAGAACCA	ACTCCTTAAAGCTGCGCAGAA
*TNFα*	Human	TCTTCTCGAACCCCGAGTGA	GGAGCTGCCCCTCAGCTT
*CXCL10*	Human	GGAAATCGTGCGTGACATTA	AGGAAGGAAGGCTGGAAGAG
*Leptin*	Human	CGGAGAGTACAGTGAGCCA	CGGAATCTCGCTCTGTCAT
*Adiponectin*	Human	CCCAAAGAGGAGAGGAA	TCAGAAACAGGACACAAC
*HSL*	Human	GCCTGGGCTTCCAGTTCAC	CCTGTCTCGTTGCGTTTGTAGT
*Loricrin*	Human	GTCTGCGGAGGTGGTTCCTCT	TGCTGGGTCTGGTGGCAGATC
*Filaggrin*	Human	CATGGCAGCTATGGTAGTGCAGA	ACCAAACGCACTTGCTTTACAGA
*Cytokeratin16*	Human	CTACCTGAGGAAGAACCACGAG	CTCGTACTGGTGACGCATCTGA
*S100A7*	Human	GCACAAATTACCTCGCCGAT	GACATTTTATTGTTCCTGGGGTC

## Data Availability

The data presented in this study are available on request from the corresponding author.

## Supplementary Materials

The following supporting information can be downloaded at: https://www.mdpi.com/article/10.3390/ijms262110428/s1.

## References

[1] Pi-Sunyer X. (2009). The Medical Risks of Obesity. Postgrad. Med..

[2] Lingvay I., Cohen R.V., le Roux C.W., Sumithran P. (2024). Obesity in adults. Lancet.

[3] Obesity and Overweight. https://www.who.int/news-room/fact-sheets/detail/obesity-and-overweight.

[4] Jo J., Gavrilova O., Pack S., Jou W., Mullen S., Sumner A.E., Cushman S.W., Periwal V. (2009). Hypertrophy and/or Hyperplasia: Dynamics of Adipose Tissue Growth. PLoS Comput. Biol..

[5] Castoldi A., Naffah de Souza C., Câmara N.O.S., Moraes-Vieira P.M. (2015). The Macrophage Switch in Obesity Development. Front. Immunol..

[6] Skurk T., Alberti-Huber C., Herder C., Hauner H. (2007). Relationship between Adipocyte Size and Adipokine Expression and Secretion. J. Clin. Endocrinol. Metab..

[7] Must A., Spadano J., Coakley E.H., Field A.E., Colditz G., Dietz W.H. (1999). The Disease Burden Associated With Overweight and Obesity. JAMA.

[8] Boehncke W.-H., Schön M.P. (2015). Psoriasis. Lancet.

[9] Zhou X., Chen Y., Cui L., Shi Y., Guo C. (2022). Advances in the pathogenesis of psoriasis: From keratinocyte perspective. Cell Death Dis..

[10] Fry L., Baker B.S. (2007). Triggering psoriasis: The role of infections and medications. Clin. Dermatol..

[11] Campione E., Cosio T., Pistoia E.S., Artosi F., Shumack R.G., Borselli C., Rivieccio A., Caputo V., Favaro M., Sorge R. (2024). Prevalence of fungal colonization among patients with psoriasis in difficult-to-treat areas: Impact of apremilast on mycotic burden and clinical outcomes. Front. Immunol..

[12] Lindegård B. (2009). Diseases Associated with Psoriasis in a General Population of 159,200 Middle-Aged, Urban, Native Swedes. Dermatologica.

[13] Hwang J., Yoo J.A., Yoon H., Han T., Yoon J., An S., Cho J.Y., Lee J. (2021). The Role of Leptin in the Association between Obesity and Psoriasis. Biomol. Ther..

[14] Dopytalska K., Baranowska-Bik A., Roszkiewicz M., Bik W., Walecka I. (2020). The role of leptin in selected skin diseases. Lipids Health Dis..

[15] He Q., Niu M., Bi J., Du N., Liu S., Yang K., Li H., Yao J., Du Y., Duan Y. (2023). Protective effects of a new generation of probiotic *Bacteroides fragilis* against colitis in vivo and in vitro. Sci. Rep..

[16] Qu D., Sun F., Feng S., Yu L., Tian F., Zhang H., Chen W., Zhai Q. (2022). Protective effects of *Bacteroides fragilis* against lipopolysaccharide-induced systemic inflammation and their potential functional genes. Food Funct..

[17] Troy E.B., Kasper D.L. (2010). Beneficial effects of *Bacteroides fragilis* polysaccharides on the immune system. Front. Biosci. J. Virtual Libr..

[18] Abouelela M.E., Helmy Y.A. (2024). Next-Generation Probiotics as Novel Therapeutics for Improving Human Health: Current Trends and Future Perspectives. Microorganisms.

[19] Al-Fakhrany O.M., Elekhnawy E. (2024). Next-generation probiotics: The upcoming biotherapeutics. Mol. Biol. Rep..

[20] Chervet A., Nehme R., Defois-Fraysse C., Decombat C., Blavignac C., Auxenfans C., Evrard B., Michel S., Filaire E., Berthon J.-Y. (2025). Development and characterization of a chicory extract fermented by *Akkermansia muciniphila*: An in vitro study on its potential to modulate obesity-related inflammation. Curr. Res. Food Sci..

[21] Jensen P., Skov L. (2017). Psoriasis and Obesity. Dermatology.

[22] Wang J., Yu Y., Liu L., Wang C., Sun X., Zhou Y., Hong S., Cai X., Xu W., Li X. (2024). Global prevalence of obesity in patients with psoriasis: An analysis in the past two decades. Autoimmun. Rev..

[23] Cohen A.D., Sherf M., Vidavsky L., Vardy D.A., Shapiro J., Meyerovitch J. (2008). Association between Psoriasis and the Metabolic Syndrome: A Cross-Sectional Study. Dermatology.

[24] Frasca D., Strbo N. (2022). Effects of Obesity on Infections with Emphasis on Skin Infections and Wound Healing. J. Dermatol. Skin Sci..

[25] Darlenski R., Mihaylova V., Handjieva-Darlenska T. (2022). The Link Between Obesity and the Skin. Front. Nutr..

[26] Zhang T., Yang Y., Liang Y., Jiao X., Zhao C. (2018). Beneficial Effect of Intestinal Fermentation of Natural Polysaccharides. Nutrients.

[27] Vallianou N.G., Kounatidis D., Tsilingiris D., Panagopoulos F., Christodoulatos G.S., Evangelopoulos A., Karampela I., Dalamaga M. (2023). The Role of Next-Generation Probiotics in Obesity and Obesity-Associated Disorders: Current Knowledge and Future Perspectives. Int. J. Mol. Sci..

[28] Bourebaba Y., Marycz K., Mularczyk M., Bourebaba L. (2022). Postbiotics as potential new therapeutic agents for metabolic disorders management. Biomed. Pharmacother..

[29] Zhu Y., Xu F., Chen H., Zheng Q. (2024). The efficacy and safety of probiotics in the adjuvant treatment of psoriasis: A systematic review and meta-analysis of randomized controlled trials. Front. Med..

[30] Rather I.A., Bajpai V.K., Huh Y.S., Han Y.-K., Bhat E.A., Lim J., Paek W.K., Park Y.-H. (2018). Probiotic *Lactobacillus sakei* proBio-65 Extract Ameliorates the Severity of Imiquimod Induced Psoriasis-Like Skin Inflammation in a Mouse Model. Front. Microbiol..

[31] Cang W., Li X., Tang J., Wang Y., Mu D., Wu C., Shi H., Shi L., Wu J., Wu R. (2024). Therapeutic Potential of *Bacteroides fragilis* SNBF-1 as a Next-Generation Probiotic: In Vitro Efficacy in Lipid and Carbohydrate Metabolism and Antioxidant Activity. Foods.

[32] Rios-Covian D., Arboleya S., Hernandez-Barranco A.M., Alvarez-Buylla J.R., Ruas-Madiedo P., Gueimonde M., de los Reyes-Gavilan C.G. (2013). Interactions between Bifidobacterium and Bacteroides Species in Cofermentations Are Affected by Carbon Sources, Including Exopolysaccharides Produced by Bifidobacteria. Appl. Environ. Microbiol..

[33] Rios-Covian D., Sánchez B., Salazar N., Martínez N., Redruello B., Gueimonde M., de los Reyes-Gavilán C.G. (2015). Different metabolic features of *Bacteroides fragilis* growing in the presence of glucose and exopolysaccharides of bifidobacteria. Front. Microbiol..

[34] Manna P., Jain S.K. (2015). Obesity, Oxidative Stress, Adipose Tissue Dysfunction, and the Associated Health Risks: Causes and Therapeutic Strategies. Metab. Syndr. Relat. Disord..

[35] Forrester S.J., Kikuchi D.S., Hernandes M.S., Xu Q., Griendling K.K. (2018). Reactive Oxygen Species in Metabolic and Inflammatory Signaling. Circ. Res..

[36] Kim S., Lee J.Y., Jeong Y., Kang C.-H. (2022). Antioxidant Activity and Probiotic Properties of Lactic Acid Bacteria. Fermentation.

[37] Kim H., Kim J.-S., Kim Y., Jeong Y., Kim J.-E., Paek N.-S., Kang C.-H. (2020). Antioxidant and Probiotic Properties of Lactobacilli and Bifidobacteria of Human Origins. Biotechnol. Bioprocess Eng..

[38] Appari M., Channon K.M., McNeill E. (2018). Metabolic Regulation of Adipose Tissue Macrophage Function in Obesity and Diabetes. Antioxid. Redox Signal..

[39] Canton M., Sánchez-Rodríguez R., Spera I., Venegas F.C., Favia M., Viola A., Castegna A. (2021). Reactive Oxygen Species in Macrophages: Sources and Targets. Front. Immunol..

[40] Son Y., Cheong Y.-K., Kim N.-H., Chung H.-T., Kang D.G., Pae H.-O. (2011). Mitogen-Activated Protein Kinases and Reactive Oxygen Species: How Can ROS Activate MAPK Pathways?. J. Signal Transduct..

[41] Chen C., He Y.-Q., Gao Y., Pan Q.-W., Cao J.-S. (2024). Extracellular vesicles of *Bacteroides fragilis* regulated macrophage polarization through promoted *Sema7a* expression. Microb. Pathog..

[42] Deng H., Li Z., Tan Y., Guo Z., Liu Y., Wang Y., Yuan Y., Yang R., Bi Y., Bai Y. (2016). A novel strain of *Bacteroides fragilis* enhances phagocytosis and polarises M1 macrophages. Sci. Rep..

[43] Wang X., Ye C., Xun T., Mo L., Tong Y., Ni W., Huang S., Liu B., Zhan X., Yang X. (2021). *Bacteroides fragilis* Polysaccharide A Ameliorates Abnormal Voriconazole Metabolism Accompanied with the Inhibition of TLR4/NF-κB Pathway. Front. Pharmacol..

[44] He F., Wu C., Li P., Li N., Zhang D., Zhu Q., Ren W., Peng Y. (2018). Functions and Signaling Pathways of Amino Acids in Intestinal Inflammation. BioMed Res. Int..

[45] Egbujor M.C., Olaniyan O.T., Emeruwa C.N., Saha S., Saso L., Tucci P. (2024). An insight into role of amino acids as antioxidants via NRF2 activation. Amino Acids.

[46] Peter K., Rehli M., Singer K., Renner-Sattler K., Kreutz M. (2015). Lactic acid delays the inflammatory response of human monocytes. Biochem. Biophys. Res. Commun..

[47] Yang H., Meng L., Ai D., Hou N., Li H., Shuai X., Peng X. (2019). Acetic acid alleviates the inflammatory response and liver injury in septic mice by increasing the expression of TRIM40. Exp. Ther. Med..

[48] Singh S.K., Kaldate R., Bisht A., Nabavi S.M., Silva A.S. (2022). Chapter4.5—Citric acid, antioxidant effects in health. Antioxidants Effects in Health.

[49] Harber K.J., de Goede K.E., Verberk S.G.S., Meinster E., de Vries H.E., van Weeghel M., de Winther M.P.J., Van den Bossche J. (2020). Succinate Is an Inflammation-Induced Immunoregulatory Metabolite in Macrophages. Metabolites.

[50] Ji Z., Feng X., Han C., Li S., Wu B., Zhang X., Zhu S., Tong W., Xu W. (2025). The malic acid inhibiting inflammation in ankylosing spondylitis by interfering M1 macrophage polarization. Int. Immunopharmacol..

[51] Hämäläinen M., Nieminen R., Vuorela P., Heinonen M., Moilanen E. (2007). Anti-Inflammatory Effects of Flavonoids: Genistein, Kaempferol, Quercetin, and Daidzein Inhibit STAT-1 and NF-κB Activations, Whereas Flavone, Isorhamnetin, Naringenin, and Pelargonidin Inhibit only NF-κB Activation along with Their Inhibitory Effect on iNOS Expression and NO Production in Activated Macrophages. Mediators Inflamm..

[52] Nakajima H., Nakajima K., Tarutani M., Morishige R., Sano S. (2011). Kinetics of circulating Th17 cytokines and adipokines in psoriasis patients. Arch. Dermatol. Res..

[53] Johnston A., Arnadottir S., Gudjonsson J.E., Aphale A., Sigmarsdottir A.A., Gunnarsson S.I., Steinsson J.T., Elder J.T., Valdimarsson H. (2008). Obesity in psoriasis: Leptin and resistin as mediators of cutaneous inflammation. Br. J. Dermatol..

[54] Kobyliak N., Conte C., Cammarota G., Haley A.P., Styriak I., Gaspar L., Fusek J., Rodrigo L., Kruzliak P. (2016). Probiotics in prevention and treatment of obesity: A critical view. Nutr. Metab..

[55] Kim Y.-K., Cho M., Kang D.-J. (2024). Anti-Inflammatory Response of New Postbiotics in TNF-α/IFN-γ-Induced Atopic Dermatitis-like HaCaT Keratinocytes. Curr. Issues Mol. Biol..

[56] Chung H.-J., Lee H., Kim M., Lee J.W., Saeed M., Lee H., Jung S.-H., Shim J.-J., Lee J.-L., Heo K. (2022). Development and metabolic profiling of a postbiotic complex exhibiting antibacterial activity against skin microorganisms and anti-inflammatory effect on human keratinocytes. Food Sci. Biotechnol..

[57] Guo J., Zhang H., Lin W., Lu L., Su J., Chen X. (2023). Signaling pathways and targeted therapies for psoriasis. Signal Transduct. Target. Ther..

[58] Pasquali L., Srivastava A., Meisgen F., Mahapatra K.D., Xia P., Landén N.X., Pivarcsi A., Sonkoly E. (2019). The Keratinocyte Transcriptome in Psoriasis: Pathways Related to Immune Responses, Cell Cycle and Keratinization. Acta Derm. Venereol..

[59] Chervet A., Nehme R., Decombat C., Longechamp L., Habanjar O., Rousset A., Fraisse D., Blavignac C., Filaire E., Berthon J.-Y. (2023). Exploring the Therapeutic Potential of Ampelopsis grossedentata Leaf Extract as an Anti-Inflammatory and Antioxidant Agent in Human Immune Cells. Int. J. Mol. Sci..

[60] Habanjar O., Maurin A.-C., Vituret C., Vachias C., Longechamp L., Garnier C., Decombat C., Bourgne C., Diab-Assaf M., Caldefie-Chezet F. (2023). A bicellular fluorescent ductal carcinoma in situ (DCIS)-like tumoroid to study the progression of carcinoma: Practical approaches and optimization. Biomater. Sci..

