# Supplementary material for: Microbial Biotransformation of Chicory by Bacteroides fragilis: In Vitro Implications for Obesity-Related Psoriasis
Source: Int J Mol Sci. 2025 Oct 27;26(21):10428. doi: 10.3390/ijms262110428 (PMC12609387; doi:10.3390/ijms262110428)
Supplement: Supplementary file 1 [file ijms-26-10428-s001.zip › ijms-3889316-supplementary.pdf]

**Supplementary Table S1 : HPLC analysis**

| | **Chicory** | ***C-B.fragilis*** |
|---|---|---|
| **Propionic acid** | / | / |
| **Butyric acid** | / | / |
| **Valeric acid** | / | / |
| **Malic acid** | 0.9403 g/L | 2.1483 g/L |
| **Lactic acid** | / | 5.3823 g/L |
| **Acetic acid** | / | 3.0849 g/L |
| **Citric acid** | 0.7487 g/L | 3.7506 g/L |
| **Succinic acid** | / | 2.1018 g/L |
